# *c-myc* copy number gain is a powerful prognosticator of disease outcome in cervical dysplasia

**DOI:** 10.18632/oncotarget.2706

**Published:** 2014-12-16

**Authors:** Kirsten Kübler, Sally Heinenberg, Christian Rudlowski, Mignon-Denise Keyver-Paik, Alina Abramian, Sabine Merkelbach-Bruse, Reinhard Büttner, Walther Kuhn, Hans-Ulrich Schildhaus

**Affiliations:** ^1^ Department of Obstetrics and Gynecology, Center for Integrated Oncology, University of Bonn, 53127 Bonn, Germany; ^2^ Evangelisches Krankenhaus, Teaching Hospital of the University of Bonn, 51465 Bergisch Gladbach, Germany; ^3^ Institute of Pathology, Center for Integrated Oncology, University of Bonn, 53127 Bonn, Germany; ^4^ Institute of Pathology, Center for Integrated Oncology, University of Cologne, 50937 Cologne, Germany; ^5^ Institute of Pathology, University of Göttingen, 37075 Göttingen, Germany

**Keywords:** cervical intraepithelial neoplasia, FISH analysis, copy number gain, prognostic signature, *c-myc*

## Abstract

Cervical carcinoma develops from preneoplasia by a multistep process. Although most low-grade dysplastic lesions will regress without intervention and even high-grade changes exhibit a substantial rate of regression, a small percentage of dysplasia will progress over time. Thus, indicators are needed to estimate the biological risk and to help avoid overtreatment in women who desire to preserve fertility. In addition to the classical biomarkers, PCR-ELISA-determined HPV genotype and immunohistochemically assessed p16^INK4a^ and Ki-67 expression, cells with integrated HPV and copy number gain of *TERC* and *c-myc* were quantified in a panel of 104 benign, intraepithelial neoplastic (CIN I, II, III) and cancerous lesions using fluorescence *in situ* hybridization. Optimal cut-off values were calculated; Kaplan-Meier curves and a Cox proportional hazard regression model were used to evaluate prognostic signatures. The assay reliably identified HPV integration, *TERC* and *c-myc* copy number gain as determined by comparisons with established biomarkers. All biomarker levels increased with the progression of the disease. However, only *c-myc* copy number gain independently prognosticated a low probability of dysplastic regression. Our results suggest that *c-myc* plays a key role in the process of dysplastic transformation and might thus be exploited for treatment and follow-up decision-making of cervical dysplasia.

## INTRODUCTION

Squamous cell carcinoma (SCC) of the cervix continues to be the fourth most common malignancy in women worldwide and the fourth leading cause of cancer-related death, even though the tumor is nowadays virtually preventable through the combination of human papillomavirus (HPV) vaccination and screening programs. The early detection system of SCC is based on the fact that the tumor develops through a well-defined continuum of premalignant lesions (cervical intraepithelial neoplasia, CIN) that can be identified and treated before malignant transformation. However, detailed insights into the natural history of SCC point out that not all untreated premalignant abnormalities would develop into cancer [1]. Even for advanced CIN the amount of non-progressing lesions appears to be considerable [2]. Yet, regardless of biological diversity standard management consists of colposcopy-directed biopsies to determine the severity of the lesion and cervical conization in the case of advanced CIN. This approach has led to a substantial rate of overtreatment that persists to be a problem in young women in which cervical excision is associated with an increased risk of adverse pregnancy outcome [3]. Thus, there is a need for biomarkers that more precisely prognosticate the natural course of the disease.

Several candidate prognostic indicators have been evaluated in the last years [4]. Most of them are conceptually based on molecular key events of HPV-associated carcinogenesis. The virus is attributed to be the cause of virtually all SCC cases [5] and promotes malignant transformation through the integration of the viral oncogenes E6/E7 into the host genome subsequently inactivating p53 and retinoblastoma (RB1) tumor suppressor [6]. As a consequence, genes required for S-phase entry get continuously activated. Two of the most thoroughly studied molecular biomarkers, the tumor suppressor gene p16^INK4a^ and the proliferation-associated gene Ki-67, reflect this deregulation of the cell cycle [7]. Uncontrolled cell growth may then lead to genomic instability. Accordingly, recurrent patterns of genetic aberrations have been observed in SCC. The most commonly identified amplifications include the chromosomal regions 3q and 8q, which coincide with the mapping positions of the telomerase RNA component (*TERC*) gene at 3q26.3 and the protooncogene *c-myc* at 8q24.2 [8]. Upregulation of both suggested candidate genes have been shown to be critical events in the progression of cervical precursor lesions to a malignant phenotype [9–13]. Consequently, *TERC* and *c-myc* copy number gains have been proposed as two of the most promising biomarkers [14].

Due to ethical limitations of follow-up research in preneoplastic changes the disease behavior according to the status of a specific biomarker is only poorly documented in the literature. We established a fluorescence *in situ* hybridization (FISH) assay that simultaneously tested for HPV integration, *TERC* and *c-myc* copy numbers in formalin-fixed paraffin-embedded (FFPE) tissue specimens. Correlation with HPV genotypes and expression of the classical biomarkers p16^INK4a^ and Ki-67 validated the FISH assay. Signatures were then tested for their potential to prognosticate the cumulative risk of persistent and progressive disease over time in a cohort of women that were followed-up and treated according to guidelines.

## RESULTS

### Performance of the FISH assay

**Figure 1:**
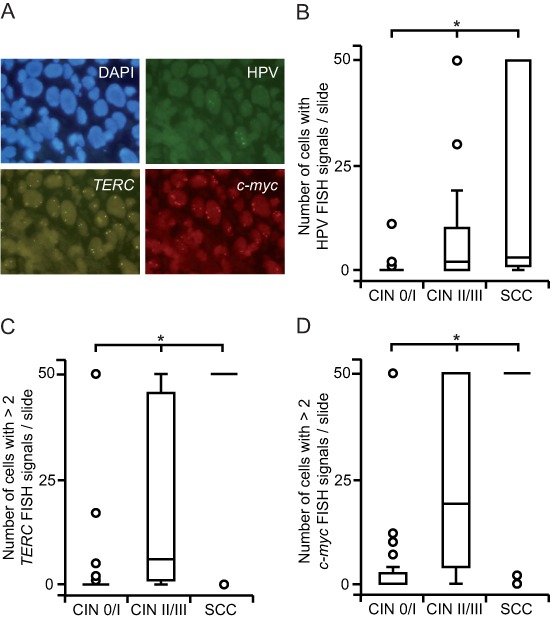
HPV integration, *TERC* and *c-myc* copy number gain increase with progressing severity of cervical lesions **(A)** FISH-visualization of integrated HPV DNA (green), *TERC* (gold) and *c-myc* (red) signals in a FFPE specimen of CIN III; nuclei of the cells are shown in blue (representative fluorescence images, 100x magnification). (**(B)** HPV FISH positive cells accumulate along the increasing severity of cervical lesions. FISH spots were counted up to a maximum of 50 signals and grouped according to histopathology. **(C, D)** The quantity of cells with *TERC* **(C)** and *c-myc* **(D)** copy number gain is higher in SCC and CIN II / III compared to CIN 0 / I cohorts. Cells with > 2 FISH signals were counted up to a maximum of 50 and classified according to histopathology. Box plots summarize the median, the 25th and 75th percentiles, the whiskers and the outliers (*p < 0.05, after Bonferroni correction).

Integrated HPV, *TERC* and *c-myc* FISH spots were of comparable intensity in HeLa cells prepared by liquid-based cytospin and tissue processing (Supplementary Fig. S1). We then applied the method to FFPE specimens of cervical changes. Fluorescent signals of integrated HPV, *TERC* and *c-myc* showed similar intensities across all tissue specimens (Fig. 1A). Successful hybridization of HPV was achieved in 98 (94%), of *TERC* in 66 (64%) and of *c-myc* in 81 (78%) cases.

### HPV genotype and integration status in relation to histopathology

Oncogenic HPV genotypes were more commonly found in SCC and higher- than lower-grade dysplastic changes (Table 1). However, ten malignant and high-grade dysplastic cases showed no evidence of high-risk HPV. Eight of these exhibited signs of integrated HPV DNA as well as *TERC* and *c-myc* copy number gain suggesting a false-negative PCR result due to HPV integration that may have disrupted PCR primer target sequences. According to HPV genotype data also the prevalence of HPV integration was more frequent in cancerous and high- than low-grade dysplastic lesions ( *p* < 0.01; Fig. 1B). Benign tissue did not show increased HPV FISH signals and only four CIN I cases harbored HPV integration events.

**Table 1:** Correlation of HPV genotypes with histopathology and the course of the disease

Variable	No, low-risk HPV	High-risk HPV	*p*-value*
	*n*		
CIN 0/I	38	3	<0.01
CIN II/III	5	36	
SCC	5	17	
Regression	11	14	n.s.
Persistence / Progression	9	22	

n.s., not significant.

* after Bonferroni correction.

### *TERC* and *c-myc* copy numbers in relation to histopathology

We evaluated average *TERC* and *c-myc* copy numbers per cell to determine the level of chromosomal instability. Moreover, this analysis allowed us to choose appropriate cut-off values for the discrimination of normal and abnormal cells. The average amount of both *TERC* ( *p* < 0.01; Supplementary Fig. S2A) and *c-myc* ( *p* < 0.01; Supplementary Fig. S2B) copy numbers per cell was found to be positively associated with the progression of the disease. However, only low-level copy number gain was observed in dysplastic specimens. Therefore, we used a threshold of > 2 FISH spots per cell to differentiate between healthy and unhealthy cells. In accordance to our observations above, the quantity of cells with a chromosomal gain of *TERC* ( *p* < 0.01; Fig. 1C) and *c-myc* ( *p* < 0.01; Fig. 1D) increased with the severity of cervical lesions. However, also four CIN I cases were defined by high *TERC* and *c-myc* copy number gain. Interestingly, further analysis showed that none of these women exhibited regression of dysplasia.

### Expression of p16^INK4a^ and Ki-67 in relation to histopathology

**Figure 2:**
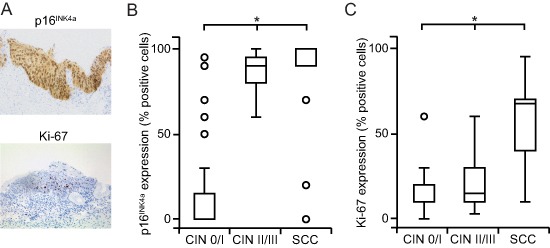
Overexpression of p16^INK4A^ and Ki-67 increases with progressing severity of cervical lesions **(A)** Cells immunohistochemically positive for p16^INK4A^ show a strong nuclear and cytoplasmic expression; cells positive for Ki-67 exhibit an intense nuclear labeling with a limited diffuse background staining (representative bright field images, 20x magnification). **(B, C)** The extent of staining for p16^INK4A^ **(B)** and Ki-67 **(C)** is higher in in SCC and CIN II / III compared to CIN 0 / I cohorts. p16^INK4a^ and Ki-67 expression was recorded as the percentage of positive cells among the total number of epithelial cells in representative areas and stratified by histopathology. Box plots summarize the median, the 25th and 75th percentiles, the whiskers and the outliers (*p < 0.05, after Bonferroni correction).

All cervical changes were stained for p16^INK4a^ and Ki-67 (Fig. 2A). We found a significant association between the expression of p16^INK4a^ and an increasing severeness of the disease ( *p* < 0.01; Fig. 2B). The upregulation of p16^INK4A^ was particularly pronounced in advanced dysplasia and SCC. Only six non-dysplastic cases displayed p16^INK4A^ expression due to squamous metaplasia. Also, the increase of Ki-67 paralleled the progression of the disease ( *p* < 0.01; Fig. 2C). However, the induction of Ki-67 appeared to be a late event during carcinogenesis and thus exhibited the most pronounced expression in SCC.

### HPV integration status, *TERC* and *c-myc* copy numbers in relation to HPV genotypes, p16^INK4A^ and Ki-67 levels

The FISH assay was further validated by analyzing for correlation between FISH results and results obtained using HPV genotypes and classical biomarkers. First, cut-off scores were determined using ROC curve analyses (Supplementary Fig. S3). Areas under the ROC curves (AUCs) for cells with *TERC* and *c-myc* copy number gain, p16^INK4A^ and Ki-67 expression obtained from the training set equaled 1.0 and thus provided a perfect separation of CIN 0 and SCC. The analysis of HPV FISH signals resulted in a lower but acceptable AUC value. In the next step, cervical changes were evaluated for concordance of HPV integration and genotype. We found that cases with carcinogenic HPV infection harbored a higher amount of HPV integration sites ( *p* < 0.01; Supplementary Fig. S4A). The accuracy of the HPV FISH analysis was further supported by the fact that HPV integration correlated with p16^INK4a^ ( *p* < 0.01; Supplementary Fig. S4B) and Ki-67 staining ( *p* < 0.01; Supplementary Fig. S4C). In accordance to these data, *TERC* copy number gain was also positively related to the presence of high-risk HPV ( *p* < 0.01; Supplementary Fig. S4D), p16^INK4a^ ( *p* < 0.01; Supplementary Fig. S4E) and Ki-67 overexpression ( *p* < 0.01; Supplementary Fig. S4F). Moreover, increased *c-myc* copy numbers were more frequent in cases defined by the presence of oncogenic HPV ( *p* < 0.01; Supplementary Fig. S4G), high p16^INK4a^ ( *p* < 0.01; Supplementary Fig. S4H) and Ki-67 levels ( *p* < 0.01; Supplementary Fig. S4I).

### Biomarker status in relation to the clinical course of cervical dysplasia

Rates of regression, persistence and progression after a 5-year follow-up period are summarized in Table 2. Risks of persistence and progression were found to increase with rising severity of dysplasia. CIN I samples had a substantial tendency to regress. In contrast, CIN III cases showed a low rate of regression. Only one case of high-grade dysplasia progressed to early SCC within a short interval and was treated with radical hysterectomy. According to guidelines, conization was a common treatment in CIN III cases. Consequently, the median follow-up time for CIN III was shorter than for CIN II and CIN I specimens.

**Table 2:** Follow-up status, period and procedure according to the grade of dysplasia

Histology	Follow-up status				Follow-up time (months)	Follow-up procedure			
	Regression	Persistence	Progression	ND		Conization	Biopsy	Cytology	ND
	n (%)				median (95% CI)	n (%)			
CIN I	11 (55)	6 (30)	1 (5)	2 (10)	8.19 (2.42 – 14)	4 (20)	4 (20)	10 (50)	2 (10)
CIN II	9 (47)	6 (32)	2 (10.5)	2 (10.5)	9.01 (0 – 19.45)	11 (58)*	2 (10.5)	4 (21)	2 (10.5)
CIN III	5 (23)	15 (68)	1 (4.5)	1 (4.5)	2.17 (1.33 – 3.01)	20 (91)	0 (0)	1 (4.5)	1 (4.5)

ND, not determined; CI, confidence interval.

* includes one case of hysterectomy.

**Figure 3:**
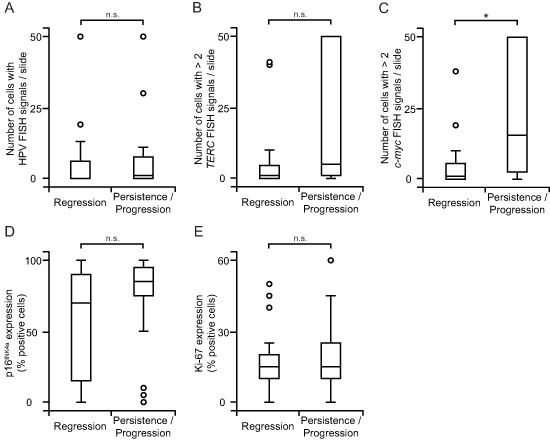
*c-myc* copy number gain correlates with persistent and progressing dysplasia **(A)** HPV integration has no value in prognosticating the course of the disease. HPV FISH signals were analyzed as in Fig. 1B and grouped according to the clinical outcome. **(B, C)** Copy numbers of *TERC* **(B)** have limited prognostic accuracy but those of *c-myc* **(C)** strongly prognosticate the course of the disease. FISH-analysis for copy number gain was performed as in Fig. 1C, D and classified according to the clinical outcome. **(D, E)** Strong expression of p16^INK4A^ and Ki-67 does not identify lesions with a high probability of persistence and progression. Analysis of p16^INK4A^ **(D)** and Ki-67 **(E)** was performed as in Fig. 2B,C and stratified according to the clinical outcome. Box plots summarize the median, the 25th and 75th percentiles, the whiskers and the outliers (*p < 0.05, after Bonferroni correction; n.s., not significant).

Oncogenic HPV genotypes were not associated with an increased risk of persistent and progressive dysplasia (Table 1). Likewise, the degree of integrated HPV DNA (Fig. 3A) as well as *TERC* copy numbers (Fig. 3B) did not prognosticate the clinical outcome. Also p16^INK4A^ (Fig. 3D) and Ki-67 (Fig. 3E) overexpression did not identify cervical changes with a high likelihood of persistence and progression. Only lesions that showed a particularly pronounced gain of *c-myc* copy numbers were more likely to persist and progress ( *p* < 0.01; Fig. 3C).

### Cumulative risk of persistence and progression of cervical dysplasia over time

**Figure 4:**
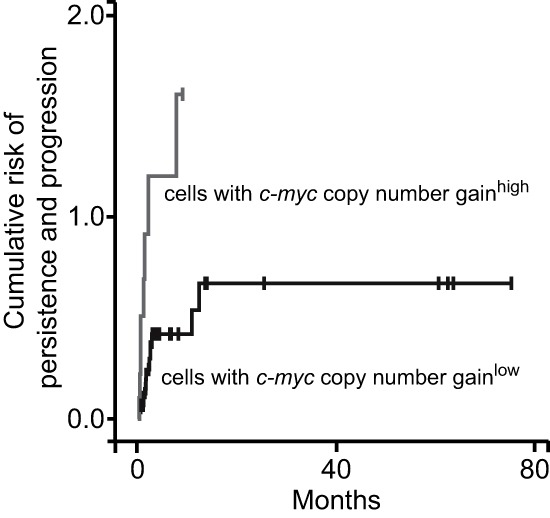
Copy numbers of *c-myc* are useful for risk prognostication in dysplastic lesions FISH-analysis of *c-myc* copy number gain was performed as in Fig. 1D; ROC curve analysis-based cut-off values defined low-risk and high-risk groups; Kaplan Meier curves according to risk tier are shown.

In the next step, we analyzed the cumulative hazard of persistence and progression over time in low- and high-risk groups. The risk of persistent and progressive disease was significantly elevated in patients with high *c-myc* copy numbers ( *p* < 0.01; Fig. 4). Of note, the cumulative hazard value was more than one-fold larger in lesions with high *c-myc* copy numbers. The analysis of the HPV genotype and integration status, *TERC* copy number gain, p16^INK4a^ and Ki-67 overexpression did not define high-risk entities with a hazard different from that of the low-risk group (data not shown).

According to our results above, univariate analysis identified only *c-myc* copy number gain as a risk factor for the persistence and progression of dysplasia (Table 3). Also in the multivariate model *c-myc* copy number gain remained significant. Thus, the level of *c-myc* copy numbers appeared to be a strong independent risk factor.

**Table 3:** Risk factors affecting persistence and progression of dysplasia

Variable	Persistence / Progression			
	Univariate analysis		Multivariate analysis	
	Hazard ratio (95% CI)	*p*-value*	Hazard ratio (95% CI)	*p*-value
HPV oncogenic genotype	1.93 (0.88 – 4.22)	n.s.	0.52 (0.13 – 2.07)	n.s.
High number of cells with HPV FISH signals / slide	1.01 (0.99 – 1.03)	n.s.	1.02 (0.99 – 1.06)	n.s.
High number of cells with > 2 *TERC* signals / slide	2.20 (0.77 – 6.26)	n.s.	0.94 (0.19 – 4.71)	n.s.
High number of cells with > 2 *c-myc* signals / slide	1.03 (1.01 – 1.06)	0.006	1.04 (1.00 – 1.08)	0.049
p16^INK4a^ overexpression (% positive cells)	1.01 (1.00 – 1.03)	n.s.	1.01 (0.99 – 1.03)	n.s.
Ki-67 overexpression (% positive cells)	1.02 (0.99 – 1.04)	n.s.	1.03 (1.00 – 1.07)	n.s.

CI, confidence interval; n.s., not significant.

* after Bonferroni correction.

## DISCUSSION

Similar to classical biomarkers FISH-based HPV integration status as well as *TERC* and *c-myc* copy numbers showed a progressive upregulation with increasing severity of cervical dysplastic lesions thereby confirming the consistency of our data set and verifying the reliability of the FISH assay. Moreover, the present study identified *c-myc* as a biomarker with a high clinical potential since it independently stratified persistent and progressive from regressing alterations.

Although we have optimized the protocol for FFPE tissue a remaining limitation of the FISH-procedure was the inability to achieve successful hybridization for *TERC* and *c-myc* in all cases. The reduced hybridization efficiency might be due to tissue preparation procedures especially long-term fixations with formalin known to be responsible for cross-linking and thus reduced probe penetration. Probe lengths might have also influenced the efficacy of DNA detection. Indeed, hybridization with the large-sized *c-myc* probe (821 kb) led to the identification of a higher number of positive cells than did the hybridization with the small-sized *TERC* probe (495 kb). However, not only in FFPE tissue but also in cytological specimens a rate of unsuccessful hybridization for TERC of 16–47% has been reported [10, 15].

In agreement with previous data, our FISH analysis revealed low-level copy number changes in dysplastic transformations [11, 12]. Also in SCC 3q26.3 and 8q24.2 copy numbers have been shown to increase only 3–7 fold [16]. The role of limited chromosomal gain in tumorigenesis is still poorly understood but accumulating evidence suggests that even the increase of one single gene copy may influence the tumor phenotype [17].

Classical biomarkers are widely used for squamous differentiation including p16^INK4a^ and Ki-67. According to previous studies, staining values of both proteins increased stepwise with rising grades of malignant transformation [18, 19]. In line with previous findings a reliable classification of cervical changes in our cohort was also possible on the basis of HPV genotypes und integration status [11, 13]. Our results of increasing *TERC* copy numbers with rising grades of cervical lesions are similarly in agreement with previous work [10, 11, 13]. The upregulation of *TERC*, which is the RNA matrix for telomere synthesis, has been identified in several cancer entities and its effects on tumor growth include both telomerase activity-dependent and -independent pathways [20]. However, it should be noted that the 3q26.3 region also contains PIK3CA that has been additionally proposed as an oncogene in SCC potentially leading to 3q amplification [21]. Additionally, our data of *c-myc* copy number gain are in line with previous findings showing an upregulation of *c-myc* parallel to increasing dysplastic transformation [9, 12, 14].

Our analysis focused on the capacity of biomarkers to provide information on the malignant potential of dysplastic changes. To date, only little information is available on rates of persistence and progression of dysplasia according to risk factors [22, 23]. We used the combination of persistent and progressing changes as a solid end point because the small number of progressing lesions in our study may have hampered any meaningful testing. This approach is also clinically reasonable because precursor changes that will persist or progress are characterized by continual HPV infection and thus need medical treatment in contrast to those lesions that are likely to have spontaneous regression [24]. The mean time for progression of CIN III to SCC has been shown to be 8.1 to 12.6 years [25]. Thus, to correctly assess the risk of progression adequately long follow-up periods are needed. In our study an extended observation interval would have been unethical since we included CIN III, which is an immediate precursor of SCC and requires medical treatment. However, the estimatation of progression in our cohort was comparable to that in other short-term follow-up reports [26–28]. Equally important for clinical practice are proportions of regression, which were found to be adequately high in our sample [1].

We found that lesions with high *c-myc* copy numbers are prone to persist and progress. To the best of our knowledge, this is the first report providing evidence that *c-myc* copy number changes have the potential to identify precursor lesions at risk. It is well documented, that *c-myc* overexpression drives malignant transformation by controlling cellular proliferation, differentiation and apoptosis [29]. Accordingly, *c-myc* has been shown to contribute to the development of several tumor types [30]. In SCC, the *c-myc* region is a hotspot of HPV integration suggesting that the virus might alter gene copy numbers at this locus thereby harnessing *c-myc* for the course of the disease [31]. Further support for this hypothesis comes from the observation that SCC cases with a more aggressive biologic behavior harbor amplifications of the *c-myc* gene [32, 33]. Moreover, the association of low-level copy number gain with aberrant protein expression underlines the functional relevance of *c-myc* amplification in SCC [16].

In contrast to recent reports we were unable to identify *TERC* as a prognostic factor [15, 34–36]. Such discrepancy may be due to the use of different material since prior studies were entirely based on cytological specimens. Additionally, in contrast to our cohort, three studies [34–36] were based on the analysis of low-grade dysplasia solely and only one study [15] used CIN I and II lesions. Also, all authors used cut-off criteria different from our values. In order to more closely reflect tumor biology we first enumerated FISH signals per cell in hotspot areas similar to HER2 analysis in routine diagnostics [37]. Based on these data the cut-off between normal and abnormal was established. Additional ROC curve analysis was used to set stringent threshold scores for further comparative analyses.

The strength of our investigation lies in the histological verification of all cervical lesions. It is well known that studies using cytology cannot provide precise estimates of disease trends over time and that tissue specimens better reflect the course of the disease [38]. However, there is debate whether biopsies may influence the natural course by completely eradicating small lesions or initiating inflammatory healing processes [39, 40]. As of the retrospective design of our study with a relatively small number of patients further prospective research is needed to confirm the prognostic role of *c-myc*.

In conclusion, FISH-based detection of HPV integration, *TERC* and *c-myc* copy number changes as well as the analysis of HPV genotypes and p16^INK4a^ and Ki-67 overexpression is suitable for a reliable differentiation of cervical changes. However, only *c-myc* copy numbers independently identified high-risk groups for disease persistence and progression making it a potential future ancillary tool in risk determination and a valuable help in the clinical decision-making process.

## METHODS

### Patients and specimens

After approval by the Institutional Review Board of the Medical faculty FFPE tissue specimens were retrospectively collected from 104 patients treated between 2004 and 2008 at the University of Bonn. Clinical parameters were assessed using medical records; follow-up data were updated until June 2013. Samples obtained by colposcopy-directed biopsy were classified according to their histopathologic diagnosis based on World Health Organization (WHO) criteria: benign squamous lesions (CIN 0, metaplasia, hyperplasia, inflammation), squamous intraepithelial lesions (CIN I, mild dysplasia; CIN II, moderate dysplasia; CIN III, severe dysplasia / carcinoma in situ), SCC. CIN I lesions were referred to as low-grade and CIN II and III as high-grade changes [41]. Due to the small sample size individual histopathological groups were pooled for comparison as reported earlier [42]. CIN changes were followed up with surgical excision or colposcopy-directed biopsy; Pap smears were only allowed in cases being colposcopically and cytologically negative for dysplasia. The median number of biopsies obtained per woman was 2 (range, 1–3). The outcome was defined as progression (CIN I to CIN II, CIN III or SCC; CIN II to CIN III or SCC; CIN III to SCC), persistence or regression (CIN I to benign; CIN II to CIN I or benign; CIN III to CIN II, CIN I or benign). Prolonged follow-up in high-grade dysplasia was made possible only because patients delayed their treatment. Detailed patient baseline characteristics are listed in Supplementary Table S1.

### Cell line

The FISH protocol was established using the human cervical cancer cell line HeLa (ATCC^®^ CCL-2™), maintained in DMEM/F12 supplemented with 10% FBS, 200 IU/ml Penicillin, 200 μg/ml Streptomycin and 1% L-Glutamine. The identity of the cell line was verified by DNA profiling (DSMZ, Braunschweig, Germany). Using a cytospin centrifuge (Thermo Scientific, Waltham, Massachusetts USA) 2.5 × 10^4^ HeLa cells were transferred to microscope slides, fixed with methanol / glacial acetic acid, air dried and stained. Alternatively, HeLa cells were fixed with 4% paraformaldehyde, suspended in 2% agar and paraffin-embedded.

### HPV typing by PCR-ELISA

Genomic and viral DNA was extracted from microdissected samples applying the QIAamp DNA Mini Kit (Qiagen, Hilden, Germany) and screened for the presence of HPV-6/-11/-16/-18/-31/-33/-35/-45/-52/-56/-58 DNA according to a published protocol [43].

### Immunohistochemical staining

Immunostaining was performed on an automated staining system (TechMate 500, Dako, Hamburg, Germany) using the streptavidin-biotin complex method. Antibodies included the mouse anti-human Ki-67 IgG_1_ monoclonal antibody (clone MIB-1, dilution 1:1000, Dako) and the CINtec^®^ Histology kit for the evaluation of p16^INK4a^ (Roche, Basel, Switzerland).

### Evaluation of immunohistochemical staining

Immunostained cells were analyzed at 20x magnification with a Leica DM LB2 microscope (Leica Microsystems, Wetzlar, Germany). Continuous variables were generated for p16^INK4a^ and Ki-67 expression. Labeling indices were computed as the percentage of positive cells among the total number of epithelial cells scored in representative areas. For Ki-67 a nuclear staining was interpreted as positive. For p16^INK4a^ a moderate or strong nuclear and cytoplasmic labeling was regarded as positive. We favored this simple method over a semiquantitative scoring system since both protocols gave similar results for the determination of p16^INK4a^ expression [44].

### FISH analysis

A FISH assay was developed for the analysis of HPV integration, *TERC* and *c-myc* copy numbers in FFPE tissue samples by modifying a previously reported method [45]. HeLa cells served as positive controls to guarantee probe specificity, sensitivity and interpretation accuracy as they show integration of HPV-18 and gene enrichment for 3q26 and 8q24 [46–48]. Cytospinned cells were pretreated for hybridization by pepsin digestion and fixed in 1% paraformaldehyde. The probe mixture was applied and hybridization was performed at 37°C overnight. Slides were then washed in 75°C 0.4xSCC / 0.3% NP-40 for 2 min followed by an ascending series of ethanol for dehydration. In FFPE specimens, the hybridization conditions were optimized by applying a harsh pretreatment, decreasing the number of post-hybridization washing steps and performing long-term proteloytic digestion. In detail, tissue sections of 2 μm were pretreated using a VP 2000 automated tissue-processing station (Abbott, Wiesbaden, Germany). Specimens were rinsed with xylene to remove paraffin, dehydrated in a descending ethanol series and incubated in 0.2 M HCl for 20 min. To increase DNA accessibility protease digestion was performed at 37°C for 90 min. After application of the probe mixture samples were denatured at 73°C for 5 min and hybridized at 37°C overnight. Subsequently, samples were washed in 45°C 2xSCC / 0.3% NP-40 for 2 min. For FISH analysis the following probes were used: a SpectrumGold^TM^-labeled 3q26 (*TERC*) probe, a SpectrumRed^TM^-labeled 8q24 (*c-myc*) probe and a biotinylated probe for HPV-16/-18/-26/-31/-33/-35/-39/-45/-51/-52/-53/-56/-58/-59/-66/-68/-82 (kindly provided by Abbott). The green fluorescent Alexa Fluor 488 tyramide (TSA^TM^ Kit #22, Molecular Probes, Invitrogen, Darmstadt, Germany) allowed visualizing the biotin-labeled DNA. Sections were further counterstained with 4,6-diamidino-2-phenylindole (DAPI). To account for potential problems of FISH in FFPE, especially with regard to the absence of probe signals and nonspecific hybridization, HeLa cells were used to confirm proper tissue treatment. In detail, the number of FISH signals in HeLa controls were counted and compared with the number of spots expected from the literature. Thus, we were able to identify the loss of target genes due to non-optimal assay conditions and to consequently repeat the assay when the correct pattern was not obtained.

### Evaluation of FISH analysis

Slides were analyzed at 100x magnification using a Leica DM5500 fluorescence microscope (Leica Microsystems) with a triple bandpass filter for simultaneous detection. The entire surface of each slide was evaluated for FISH analysis; in cases with a high amount of FISH signals only the first 50 spots or abnormal cells were recorded. For the determination of HPV integration cells with ≥ 1 punctate HPV FISH signal localized to the nucleus indicative of HPV integration were quantified; a diffuse nuclear pattern that constitutes episomal HPV was excluded from quantification. For the determination of *TERC* and *c-myc* copy number gain two consecutive analyses were performed. First, we enumerated *TERC* and *c-myc* FISH signals in 20 cells of hotspot areas defined as the region where the most atypical cells were found. In the next step, the average number of *TERC* and *c-myc* signals per cell was assessed. From these data, the cut-off between normal and abnormal was achieved (> 2 FISH spots / cell). Using this threshold cells with *TERC* and *c-myc* copy number gain were enumerated in the whole slide.

### Statistical analysis

Comparisons between continuous data were performed using the Mann-Whitney U test; comparisons between categorical variables were carried out using the Fisher's exact test; *p-*values for tests with only a small number of counts were computed based on Monte Carlo simulations using 1.000.000 replicates. The cumulative risk of persistence or progression was assessed using the Kaplan-Meier method as a function of the length of the follow-up; the log-rank test was used to determine a statistical significance of differences. To define cut-off values samples were divided into two subsets, the training and the validation set. The former consisted of benign and SCC samples and was used to detect optimal thresholds by receiver operator characteristics (ROC) curve analysis. The precancerous samples were used separately to validate the cut-off points identified by the training set. To study the simultaneous effect of prognostic factors multivariate analysis was performed using the Cox proportional hazard regression model. The median follow-up time was calculated using the reverse Kaplan-Meier estimator [49]. Results with a two-sided *p-*value of <0.05 were considered to be significant. Conservative Bonferroni correction for *p-*values was used to account for multiple testing. Analysis was performed using the statistical softwares SPSS version 21 (IBM, Ehningen, Germany) and ‘R’ version 2.15.1 (The R Foundation for Statistical Computing, Vienna, Austria).

## SUPPLEMENTARY FIGURES AND TABLE


