# Supplementary material for: c-myc copy number gain is a powerful prognosticator of disease outcome in cervical dysplasia
Source: Oncotarget. 2014 Dec 16;6(2):825–35. doi: 10.18632/oncotarget.2706 (PMC4359258; doi:10.18632/oncotarget.2706)
Supplement: Supplementary file 1 [file oncotarget-06-825-s001.pdf]

SUPPLEMENTARY FIGURES AND TABLE

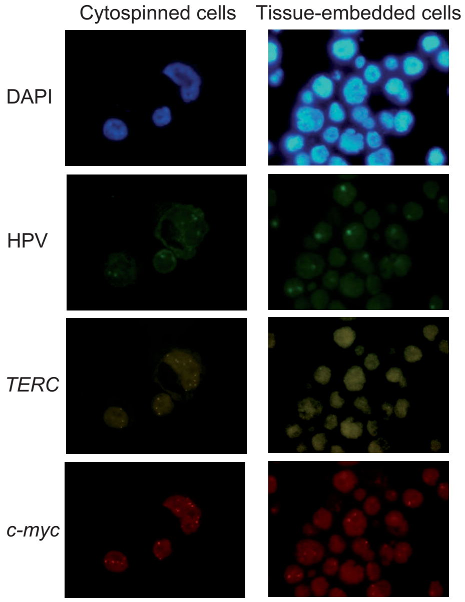

Supplementary Figure S1: FISH assay performance in HeLa cells. FISH-visualization of integrated HPV DNA (green), *TERC* (gold) and *c-myc* signals (red) in HeLa cells prepared by cytospin and tissue processing; nuclei of the cells are shown in blue (representative fluorescence images, 100x magnification).

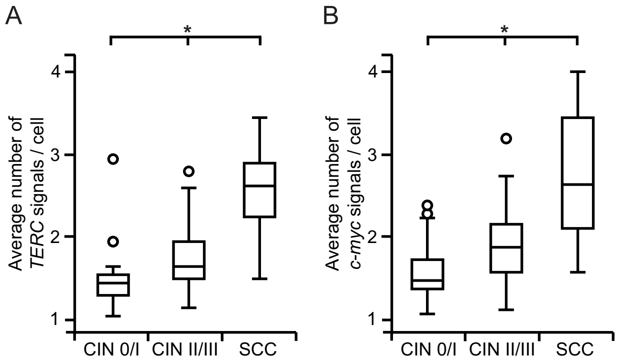

Supplementary Figure S2: *TERC* and *c-myc* copy numbers per cell increase with the progression of the disease. (A, B) FISH-analysis for *TERC* **(A)** and *c-myc* **(B)** copy numbers was performed by recording fluorescent signals in 20 cells in hotspot areas; the average number of signals per cell was determined and classified according to histopathology. Box plots summarize the median, the 25th and 75th percentiles, the whiskers and the outliers (*$p < 0.05$, after Bonferroni correction).

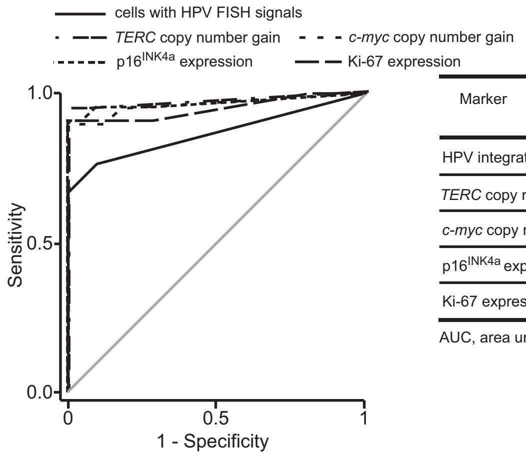

Marker	AUC	Cut-off value	Sensitivity (%)	Specificity (%)
HPV integration	0.87	≥ 1.5 cells with HPV FISH signals / slide	66.7	100
TERC copy number gain	0.97	≥ 26 cells with > 2 FISH signals / slide	94.4	100
c-myc copy number gain	0.96	≥ 26.5 cells with > 2 FISH signals / slide	90.0	100
$p16^{INK4a}$ expression	0.97	≥ 80 % positive cells	86.4	100
Ki-67 expression	0.95	≥ 25 % positive cells	90.9	100

AUC, area under the ROC curve.

Supplementary Figure S3: Definition of cut-off values for HPV FISH signals, *TERC* and *c-myc* copy number gain, $p16^{INK4A}$ and Ki-67 expression. ROC curve analysis was performed using the training set; SCC samples were used to determine sensitivity, benign lesions to determine specificity. Optimal thresholds that provide the best combination of sensitivity and specificity were selected (highest point on the vertical axis and furthest point to the left on the horizontal axis).

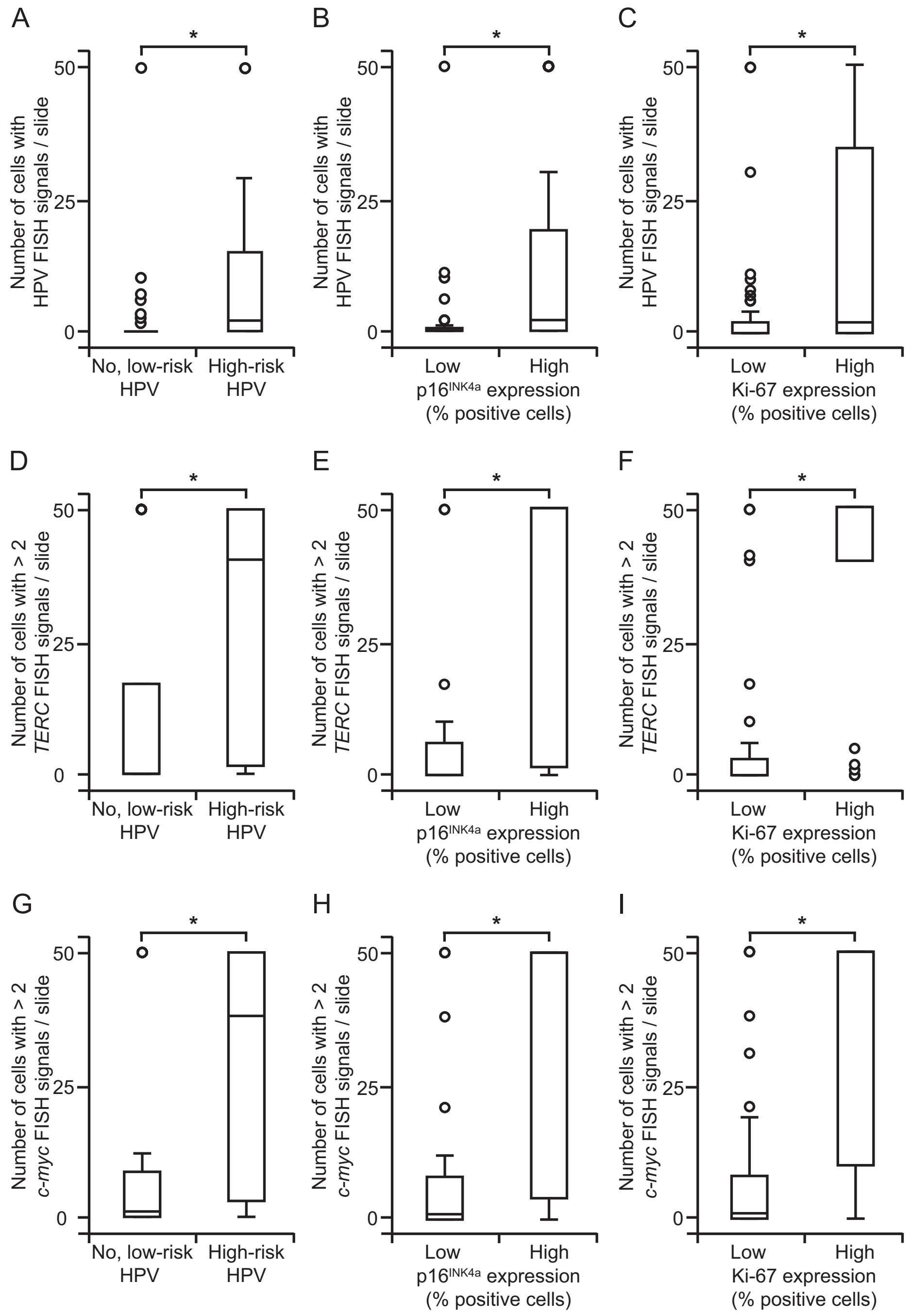

Supplementary Figure S4: Validation of the FISH assay using standard risk markers of cervical lesions. (A, B, C) FISH-based HPV integration is associated with carcinogenic HPV genotypes **(A)**, p16^{INK4a} **(B)** and Ki-67 **(C)** overexpression. FISH spots were counted up to a maximum of 50 signals in cervical lesions and grouped according to results obtained from PCR-ELISA or high and low staining levels; p16^{INK4a} and Ki-67 were determined immunohistochemically; ROC curve analysis was used to determine cut-off points. **(D, E, F)** *TERC* copy number gain is related to high-risk HPV genotypes **(D)**, p16^{INK4a} **(E)** and Ki-67 **(F)** overexpression. Cells with > 2 FISH signals were counted up to a maximum of 50 and classified as in A, B, C; p16^{INK4a} and Ki-67 were determined as in B and C; cut-off values were established as in A, B, C. **(G, H, I)** *c-myc* copy number gain is correlated to oncogenic HPV genotypes **(G)**, p16^{INK4a} **(H)** and Ki-67 **(I)** overexpression. FISH-positive cells were enumerated and classified as in D, E, F; p16^{INK4a} and Ki-67 were determined as in B and C; cut-off values were established as in A, B, C. Box plots summarize the median, the 25th and 75th percentiles, the whiskers and the outliers (*$p < 0.05$, after Bonferroni correction).

Supplementary Table S1: Clinicopathological patient characteristics

**Variable**		**Value** [median ± MAD (range)]
Age (years)	All lesions	36 ± 13.34 (21 – 80)
	CIN 0	41 ± 8.9 (24 – 60)
	CIN I	33 ± 9.64 (21 – 52)
	CIN II	28 ± 5.93 (22 – 42)
	CIN III	33.5 ± 9.64 (24 – 51)
	SCC	55.5 ± 14.83 (27 – 80)
**Variable**		**Value** [median (95% CI)]
Follow-up time (months)		9.01 (1.81 – 16.21)
**Variable**		**Value** [n (%)]
Histopathology	CIN 0	21 (20.19)
	CIN I	20 (19.24)
	CIN II	19 (18.27)
	CIN III	22 (21.15)
	SCC	22 (21.15)
Disease status	Regression	25 (40.98)
	Persistence	27 (44.26)
	Progression	4 (6.56)
	Lost to follow-up	5 (8.2)

MAD, median absolute deviation; CI, confidence interval;
CIN, cervical intraepithelial neoplasia; SCC, squamous cell carcinoma.
